# Monocyte Metabolism and Function in Patients Undergoing Cardiac Surgery

**DOI:** 10.3389/fcvm.2022.853967

**Published:** 2022-07-13

**Authors:** Daniel Mayer, Marc Altvater, Judith Schenz, Rawa Arif, Matthias Karck, Florian Leuschner, Markus A. Weigand, Florian Uhle, Christoph Lichtenstern

**Affiliations:** ^1^Department of Anesthesiology, Heidelberg University Hospital, Heidelberg, Germany; ^2^Department of Cardiac Surgery, Heidelberg University Hospital, Heidelberg, Germany; ^3^Department of Cardiology, Angiology and Pneumology, Heidelberg University Hospital, Heidelberg, Germany

**Keywords:** monocytes, cardiac surgery, immunometabolism, immune reaction, inflammation, Warburg effect

## Abstract

**Objective:**

Cardiopulmonary bypass (CPB) can lead to systemic inflammation, which is associated with higher morbidity. Therefore, we investigated the metabolism of isolated blood monocytes before and after CPB compared to healthy controls.

**Methods:**

In this prospective, monocentric, observational study, we included 30 patients undergoing CPB and 20 controls. We isolated monocytes from heparinized blood and investigated their metabolism by using Seahorse technology before (t0), 4 h (t4), and 24 h (t24) after the start of the CPB. We also examined programmed cell death 1 ligand (PD-L1), PD-L2, V-domain Ig suppressor of T cell activation (VISTA), and human leukocyte antigen-DR isotype (HLA-DR) using fluorescence-activated cell sorting analysis. Additionally, we investigated plasma cytokine levels in patients without and after *ex vivo* stimulation.

**Results:**

CPB-induced inflammatory responses are shown by significantly elevated plasma interleukin-6 levels in the CPB group compared to baseline and controls [t0: 0 ng/ml (95%CI 0-0 ng/ml); t4: 0.16 ng/ml (95%CI 0.1-0.197 ng/ml), *p* < 0.0001; t24: 0.11 ng/ml (95% CI 0.1-0.16 ng/ml), *p* < 0.0001, and controls: 0 ng/ml (95% CI 0-0 ng/ml)]. The cytokine release in the ex vivo stimulation is reduced for lipopolysaccharide stimulation at t4 [t0: 35.68 ng/ml (95% CI 22.17-46.57 ng/ml) vs. t4: 15.02 (95% CI 10.25-24.78 ng/ml), *p* < 0.0001]. Intracellular metabolism of monocytes after CPB showed a protracted shift to aerobic glycolysis [t0: 179.2 pmol/min (95% CI 138.0-205.1 pmol/min) vs. t24: 250.1 pmol/min (95% CI 94.8-300.2 pmol/min), *p* < 0.0001]. Additionally, we observed an altered metabolism in monocytes in patients undergoing cardiac surgery compared to controls even before any surgical procedure [t0: 179.2 pmol/min (95% CI 138.0-205.1) vs. controls 97.4 (95% CI 59.13-144.6 pmol/min), *p* = 0.0031].

**Conclusion:**

After CPB, patients' monocytes show a shift in metabolism from oxidative phosphorylation to aerobic glycolysis, which is associated with energy-demanding and proinflammatory processes. This is the first study to show changes in monocyte immunometabolism in cardiac surgery. Monocytes of patients undergoing cardiac surgery were leaning toward aerobic glycolysis even before any surgical procedure was conducted. Leaving the question of the pathophysiological mechanisms for future studies to be investigated and paving the way for potential therapy approaches preventing inflammatory effects of CPB.

## Introduction

For many cardiac surgery procedures, the usage of cardiopulmonary bypass (CPB) is implemented. Various mechanisms during surgery, including the CPB, cause an immune response, which can be recognized by an increase in plasma cytokines [such as interleukin (IL)-1β, IL-6, IL-8, tumor necrosis factor-alpha (TNF-α)] and activation of the complement system (C3a and C5a) (1–4).

The trauma of surgery releases intracellular and tissue proteins [damage-associated molecular patterns (DAMPs)], which activate the innate and acquired immune system (5). Besides the surgical trauma, a transient aortal clamping induces an inflammatory response by transient ischemia, which causes cell damage and an oxidative stress reaction (6). The immunological responses are partly caused by the CPB itself (7), as shown by preventing activation of the complement system in patients undergoing off-pump coronary artery bypass graft (8). The contact between blood and the nonphysiological surface of the CPB tubes leads to an activation of immune cells (9). Also, the nonpulsatile systemic blood flow, with an increased shear stress and a decreased endothelial NO synthesis, influences the immune response (10). Another potential cause of oxidative stress is hyperoxia during and after the CPB to prevent hypoxemia (11). This also indicates that CPB is one factor that increases oxidative stress (12). In addition, common surgical events affect the immune system, such as blood loss, transfusion, body temperature, duration of surgery, and others (10). Activation of the immune system after surgery is quite physiological, e.g., following tissue damage (5). The response can be local, such as an influx of monocytes and neutrophils with the release of cytokines. However, cardiac surgery using CPB can cause systemic inflammation, which is associated with higher morbidity. A systemic reaction in terms of a systemic inflammatory response syndrome (SIRS) can lead to a multi-organ dysfunction syndrome (MODS) (5).

In recent years, immunometabolism has gained more and more interest, providing a deeper understanding of metabolic pathways in immune cells being linked to their functionality (13, 14). The Warburg effect, first described in tumor cells by Otto Warburg (15), is a well-known change of intracellular metabolism to aerobic glycolysis, which also occurs in immune cells, such as monocytes. Monocytes are bone marrow-originated leukocytes and play an important role after tissue damage and during infections (16, 17). Before being activated, monocytes are primarily using oxidative phosphorylation (OXPHOS) for efficient production of energy. Inflammatory stimuli can activate monocytes and their metabolism shifts to the faster but less efficient glycolysis, despite the presence of oxygen (Warburg effect) (13, 14). Immune cells using aerobic glycolysis are known to be proinflammatory, thereby making use of the faster glycolysis to match high-energy demands during activation (18).

With the knowledge of immunometabolism, we took a closer look at the function and metabolism of monocytes in patients undergoing cardiac surgery using CPB. This is the first time monocyte immunometabolism has been assessed in cardiac surgery. We examined the metabolism of blood monocytes before, 4 h, and 24 h after CPB compared to healthy controls. We hypothesize a metabolic shift to glycolysis in monocytes after CPB according to the Warburg effect. Additionally, we measured the cytokine plasma level, the cytokine release after *ex vivo* full-blood stimulation, and surface receptor expression [programmed cell death 1 ligand (PD-L1), PD-L2, V-domain Ig suppressor of T cell activation (VISTA), and human leukocyte antigen-DR isotype (HLA-DR)] to determine their functionality. As HLA-DR has shown changes after cardiac surgery and CPB, other surface markers, such as PD-L1, which are also involved in immunometabolism, need a closer look in this field (19, 20).

## Methods

### Study Design

This is a prospective, monocentric, noninterventional observational study. We report the results of an exploratory pilot study to evaluate the occurrence of metabolic changes in monocytes over the course of cardiac surgery. With no predecessor study available, we conducted convenience sampling adapted for number of patients in our center, aiming to understand effect size and statistical estimates for upcoming studies.

We included 30 adult patients undergoing elective cardiac surgery with CPB and 20 healthy individuals without surgery or anesthesia as a control group, matched for age and gender. Participants of both groups were excluded, having an infectious viral disease, a diagnosed autoimmune disease, diabetes mellitus, pharmacologically-induced immunosuppression, mitochondrial diseases, or pregnancy. Participants of the control group were not included if they show signs of current illness of infectious origin (e.g., rhinopharyngitis), recent injuries, or recent extensive dental or surgical procedures.

The study was approved by the ethics committee of the medical faculty of the Heidelberg University, and each participant was fully informed and gave written consent before inclusion (Az: S-112/2018).

In patients undergoing cardiac surgery, blood samples were collected from the arterial line at three time points, namely, (t0) after induction of general anesthesia before surgery, (t4) 4 h after the start of CPB, and (t24) 24 h after the start of CPB. In the control group, blood was collected once after peripheral venipuncture.

### Isolation of Peripheral Blood Mononuclear Cells

Heparinized blood was centrifuged at 1,200 × *g* for 5 min to separate the plasma from the cellular parts of the blood. Plasma was removed and stored in aliquots at −80°C until further analysis. The residual cellular fraction was diluted with Dulbecco's phosphate-buffered saline solution (DPBS) (Thermo Fisher Scientific, Welham, USA), transferred to prefilled Leucosep™ density gradient centrifugation tubes, and centrifuged at 800 × *g* for 15 min without break. After removing the thrombocyte-containing plasma, the PBMCs were aspirated and washed a total of three times with isolation buffer [500 ml DPBS, Thermo Fisher Scientific, Welham, USA; 0.5% bovine serum albumin (BSA), Carl Roth GmbH + Co. KG, Karlsruhe, Germany; 2 mM ethylenediaminetetraacetic acid (EDTA), Sigma-Aldrich Chemie GmbH, München, Germany) (first centrifugation 500 × *g* for 5 min, second and third centrifugation 300 × *g* for 5 min)].

### Isolation of CD14^+^ Monocytes

To purify the CD14^+^ cells, we used the magnetic-activated cell sorting (MACS) method based on CD14-specific antibodies conjugated to magnetic beads (Miltenyi Biotec, Bergisch Gladbach, Germany). Separation was performed according to the instructions of the manufacturer. The final positive cell sorting was performed with an autoMACS Pro (Miltenyi Biotec, Bergisch Gladbach, Germany) using the “possel” program. The purity after sorting was checked by fluorescence-activated cell sorting (FACS) analysis.

### Metabolic Analysis of Monocytes

The metabolic state of isolated monocytes was determined using the Seahorse® technology (Agilent Technologies, Santa Clara, USA) on a Seahorse XFp analyzer and the Seahorse Wave Desktop Software (version 2.4). The technology is capable of measuring the extracellular acidification rate (ECAR) and oxygen consumption rate (OCR) of living cells in real-time.

To ensure proper attachment of monocytes, the corresponding culture plates were coated with Cell-Tak™ (Corning, New York, USA). Every well was incubated with 25 μl of the coating mixture (250 μl 0.1 M NaHCO_3_, pH = 8, and 4.7 μl Cell-Tak) for 20 min at room temperature (RT) with a closed lid, followed by removal of the solution and two washes with sterile water. After drying, it was stored overnight at RT and closed lid.

A total of 150,000 monocytes were used in each well for metabolic analysis in a total of 180 μl Seahorse medium (Seahorse XF base medium, Agilent Technologies, Waldbronn, Germany), 10 mM glucose, 1 mM pyruvate, 2 mM glutamine, 5 mM 4-(2-hydroxyethyl)-1-piperazineethanesulfonic acid (HEPES) at pH 7.4 ± 1. After seeding, plates were centrifuged at 300 × *g* for 1 min without a brake to accelerate cellular attachment. Before measurement, the plate was stored in a non-CO_2_ incubation chamber for 1 h. Analysis was performed using the Seahorse XFp Cell Mito Stress Kit and the Seahorse XFp Glycolytic Rate Assay Kit (both Agilent Technologies, Santa Clara, USA) according to the instructions of the manufacturer. Both kits are based on the sequential addition of different inhibitors [Oligomycin, FCCP (carbonyl cyanide-p-trifluoromethoxyphenylhydrazone), Rotenone/Antimycin A, 2-deoxy-d-glucose] to the cultured cells and the assessment of resulting changes in ECAR and OCR. In general, all tests were performed according to the instructions of the manufacturer. The Mito Stress test provided the parameters of basal respiration, adenosine triphosphate (ATP) production (from oxygen), spare respiratory capacity, and proton leak. From the glycolytic rate test, basal glycolysis and compensatory glycolysis were calculated.

### Flow Cytometry

All cellular analyses were run on a FACSVerse flow cytometer (BD Biosciences, San Jose, USA) with BD FACSuite™ software (version 1.0.5).

#### Measurement of Isolation Purity

**Figure 1:**
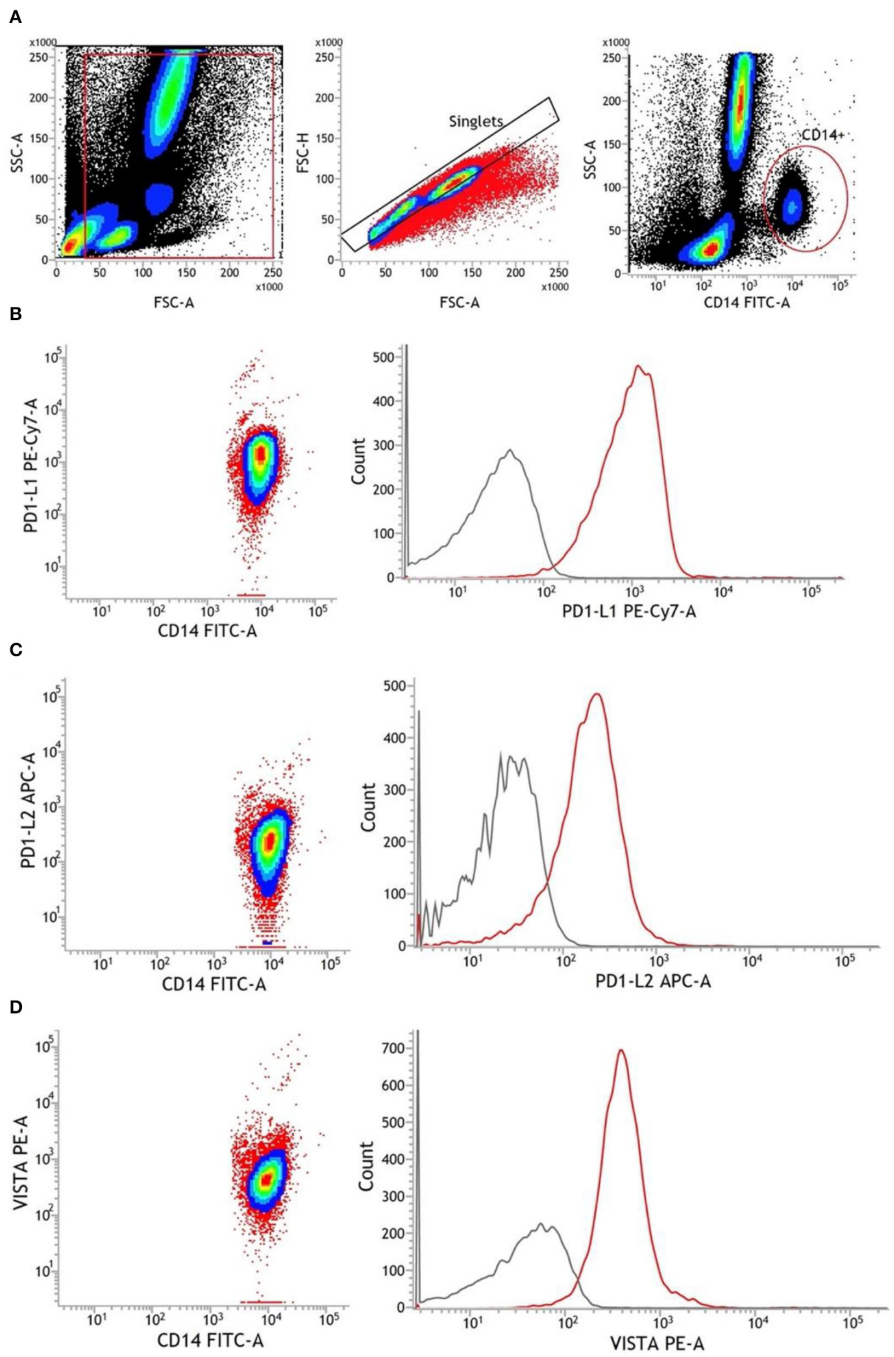
Representative gating strategy for monocytes and antibody staining of surface markers. **(A)** Chronological from left to right: debris were excluded from all cellular events. Afterward, single cells were identified *via* FSC-A and FSC-H. Using CD14 FITC antibodies, monocytes were finally detected as CD14^+^. **(B–D)** Antibody staining of PD-L1, PD-L2, and VISTA in CD14^+^ monocytes. The gray line represents FMO, and the red line represents stained monocytes. APC, allophycocyanin; FMO, fluorescence minus one; FSC-A, forward scatter area; FSC-H, forward scatter height; PE, phycoerythrin; SSC-A, side scatter area.

After isolation of CD14^+^ monocytes, 100 μl of the cell solution was mixed with 5 μl Human TruStain FcX™ (BioLegend, San Diego, USA) and incubated for 10 min to block unspecific Fc receptor bindings. Thereafter, we added 20 μl fluorescein isothiocyanate (FITC) anti-CD14 antibodies (clone: M5E2) (BD Biosciences, Heidelberg, Germany), followed by mixing and incubation for 15 min at RT in the dark. Then, the suspension was washed with 2 ml of FACS Flow solution (BD Biosciences, San Jose, USA) and centrifuged at 300 × *g* at RT for 5 min. The supernatant was removed and the pellet was resuspended in 300 μl FACSFlow. Gating was adjusted first on the cellular events in forward scatter (FSC) and side scatter (SSC), excluding particles smaller than 3 μm, followed by the assessment of CD14^+^ cells (Figure 1). Purity referred to all cellular events was calculated. All samples reached purities (fraction of CD14^+^ cells from all cells) of >80%.

#### Surface Staining and FACS Analysis of PD-L1, PD-L2, and VISTA

A total of 100 μl heparinized blood was used for each staining. Then, 5 μl Human True Stain FcX™ was added to each tube and vortexed, followed by incubation for 10 min at RT. Later, we stained the cells with Human VISTA/B7-H5/PD-1H PE-conjugated antibodies (clone: 730804; R&D Systems, Minneapolis, USA), PE-Cy™7 Mouse Anti-Human CD274 (clone: MIH1; BD Biosciences, Heidelberg, Germany), APC anti-human CD273 (B7-DC, PD-L2) (clone: MIH18; BioLegend, San Diego, USA), and FITC anti-CD14 (clone: M5E2, BD Biosciences, Heidelberg, Germany). Incubation was performed for 30 min at 4°C in the dark. Subsequent erythrocyte lysis was achieved by adding 2 ml FACS Lysing Solution (BD Biosciences, San Jose, USA) and incubation for 15 min in the dark. Before analysis, cells were centrifuged (5 min at 300 × *g*, RT), washed once with 2 ml FACS Cell Wash (BD Biosciences, San Jose, USA), and resuspended in 300 μl FACSFlow.

First, single-cellular events were gated *via* FSC and SSC to exclude debris and cellular aggregates. Then, CD14^+^ monocytes were identified and the mean fluorescence intensity (MFI) of surface markers was assessed. The CD14-FITC staining was conducted to identify CD14^+^ monocytes. Fluorescence minus one (FMO) controls were used to determine background fluorescence levels. Expression levels of PD-L1, PD-L2, and VISTA were finally measured in ΔMFI (MFI minus background fluorescence levels) (Figure 1).

#### Quantification of Monocyte HLA-DR Expression

We used 50 μl heparinized blood per staining. In every tube, we added 20 μl of Anti-Human HLA-DR antibodies (antibody mix of anti-CD14-PerCP-5.5 and anti-HLA-DR-PE; clone: L243/MϕP9; BD Biosciences, San Jose, USA). After mixing, an incubation of 30 min at RT in the dark was performed. Subsequently, 450 μl FACS Lysing Solution was added, mixed, and incubated for additional 15 min to lyse the erythrocytes. The following procedure was conducted with Anti-Human HLA-DR PE/Monocyte PerCP-Cy™5.5 (BD Biosciences, San Jose, USA) according to the protocol of the manufacturer. As each HLA-DR antibody is attached to one molecule of fluorescence dye, the obtained MFI reflects the mean number of HLA-DR-molecules per monocyte.

### *Ex vivo* Stimulation

The *ex vivo* stimulation was conducted in a 96-well TC plate (Sarstedt, Nümbrecht, Germany). Six wells were each filled with 150 μl heparinized blood and 150 μl prewarmed medium (RPMI 1640 Medium, GlutaMAX™ Supplement, HEPES; Thermo Fisher Scientific, Waltham, USA). The first two control wells were not stimulated. The third and fourth were each stimulated with 100 ng/ml ultrapure lipopolysaccharide (LPS). The last two wells were exposed to 100 μg/ml depleted zymosan. The whole plate was stored in a CO_2_ incubator (5% CO_2_, 37°C). After 24 h, the plate was centrifuged (5 min at 1,200 × *g*, RT) to obtain supernatant for further cytokine analyses.

### Enzyme-Linked Immunosorbent Assay

Analyses of the cytokines IL-6,−8, and−10 were performed using the respective DuoSet® enzyme-linked immunosorbent assay (ELISA) and DuoSet® ELISA Ancillary Reagent Kits 2 (Bio-Techne, Minneapolis, USA) following the user manual.

### Statistics

The visualization of the data and statistical analysis was performed using GraphPad Prism (version 8.0.1, GraphPad Software, La Jolla, USA). Exact Fisher's test was used for categorical data. Measurements from repeated sampling were tested by the Friedman test for global difference; if significant, Dunn's test for multiple testing between groups was performed, yielding adjusted *p*-values. For comparison of healthy controls to different time points of patients, the Kruskal-Wallis test for global differences among independent samples was performed, followed again by Dunn's post-test. Results were visualized as median and whiskers spanning the 5th and 95th percentile. *p* < 0.05 were considered significant.

## Results

### Demography

We examined 30 patients undergoing cardiac surgery and CPB and 20 healthy controls (Table 1). There was no significant difference in age between the two groups (*p* = 0.095).

**Table 1:** Group characteristics.

**Characteristics**	**Patients**	**Controls**	***p*-value**
*N*	30	20	-
Age (Years)	61.77 ± 10.8	56.55 ± 10.31	0.095
Sex (m/f)	25/5	17/3	>0.9999
BMI	26.95 (23.32–29.83)	25.47 (23.38–28.52)	0.1847
ASA-Classification 3/ 4	27/3	-	-
Duration of surgery (min)	238.5 (213.8–265.3)	-	-
CPB-duration (min)	120.5 (95.75–139)	-	-
Typ of surgery:			-
CABG-surgery	14	-	
Valve-surgery	12	-	
AVR/-R	11	-	
MVR	1	-	
Combination-surgery	4	-	-
Primary diagnosis			-
CVD	24	0	
Valvular diseases	16	0	
Foramen ovale	2	0	
Aortic aneurysm or -dissection	7	0	
Arrhythmia	7	0	
Arterial hypertonia	22	7	
Secondary diagnosis			-
Thyroid gland disorders	3	1	
COPD	1	0	
Carotid plaques	15	0	
Asthma bronchial	2	0	
Alcohol abuse	5	0	
Nicotinic abuse	10	0	
Others	8	0	
Medication			-
Analgesics	18	0	
Antihistamincs	1	0	
Anticoagulants	9	0	
β_2_-Mimetics	3	0	
Diuretics	24	6	
Cardio-circulatory drugs	24	1	
Thyroid gland hormones	3	1	
Statins	21	0	
others	12	0	

*N in absolute counts; surgery duration (cut to wound closure); age, shown as mean and standard deviation; m, male; f, female; BMI, body mass index; ASA, American Society of Anesthesiologists; CABG, coronary aortal bypass graft; AVR/-R, aortic valve replacement/repair; MVR, mitral valve repair; CVD, coronary vascular disease; valvular disease, stenose/insufficient ≥ II degree; COPD, chronic obstructive pulmonary disease*.

### Leukocytes

**Figure 2:**
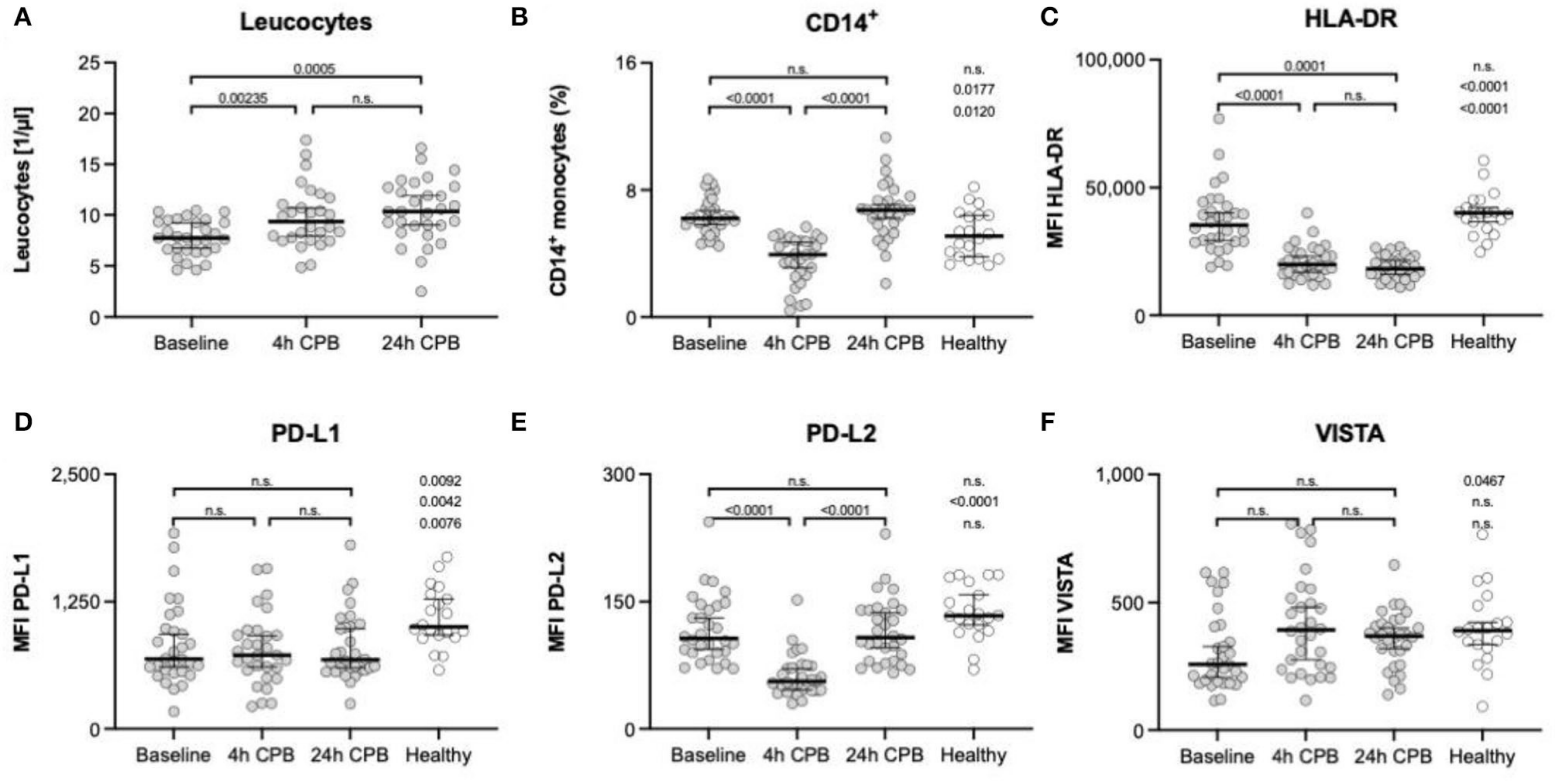
Leukocytes, CD14^+^ monocytes, and surface markers HLA-DR, PD-L1, PD-L2, and VISTA. Leukocyte count of patients **(A)** and the percentage of CD14^+^ monocytes **(B)** in full blood; FACS analyses of surface marker: **(C)** expression of HLA-DR on isolated blood monocytes; **(D)** expression of PD-L1 on isolated blood monocytes; **(E)** expression of PD-L2 on isolated blood monocytes; **(F)** expression of VISTA on isolated blood monocytes; all measurements in **(C–F)** were taken from isolated blood monocytes before surgery (baseline), 4 h after CPB (4 h CPB), 24 h after CPB (24 h CPB), and compared to the healthy control group; data shown as median and the whiskers of the 5th and 95th percentile; MWU test was used to test for significance; CPB, cardiopulmonary bypass; FACS, fluorescence-activated cell sorting; HLA, human leukocyte antigen system; ΔMFI, delta mean fluorescence intensity; PD-L, programmed cell death ligand; VISTA, V-domain Ig suppressor of T-cell activation.

While leukocytes significantly increase over time, the proportion of CD14^+^ monocytes shows a transient dip 4 h after CPB, with a rapid recovery to baseline values within 24 h after CPB (Figure 2).

### Metabolism

**Figure 3:**
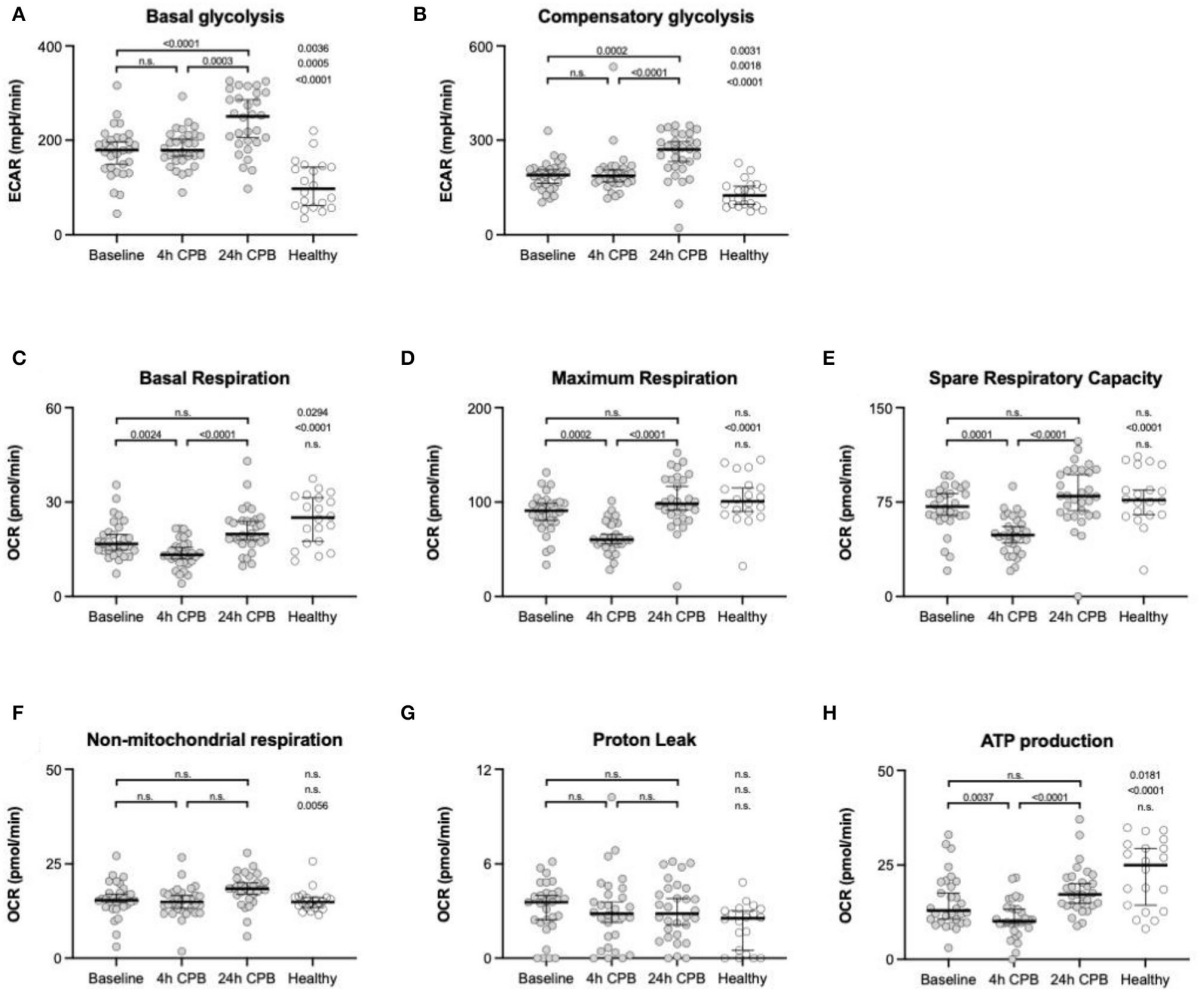
Analyses of metabolic flux. Basal glycolysis **(A)**, compensatory glycolysis **(B)**, basal respiration **(C)**, maximum respiration **(D)**, spare respiratory capacity **(E)**, non-mitochondrial respiration **(F)**, proton leak **(G)**, and ATP production **(H)** were taken from isolated blood monocytes before surgery (baseline), 4 h after CPB (4 h CPB), 24 h after CPB (24 h CPB), and compared to monocytes of the healthy control group; data shown as median and the whiskers of the 5th and 95th percentile; MWU test was used to test for significance; ATP, adenosine triphosphate; CPB, cardiopulmonary bypass; OCR, oxygen consumption rate; PER, proton efflux rate.

ATP production in healthy individuals was significantly higher than in patients (24.97 vs. 12.05 pmol/min; *p* = 0.0181) (Table 2, Figure 3H). ATP production during cardiac surgery decreased (t0 12.05 pmol/min to t4 10.13 pmol/min; *p* = 0.0024) and thereafter increased again to 17.26 pmol/min after 24 h. The basal glycolysis of healthy individuals was lower than in patients (97.4 vs. 179.2 pmol/min; *p* = 0.0035). It rose from baseline to t24 from 179.2 pmol/min to 250.1 pmol/min (*p* < 0.0001) (Table 2, Figure 3A). The compensatory glycolysis was elevated in patients undergoing cardiac surgery compared with healthy individuals (189.1 vs. 124.8 pmol/min; *p* = 0.0031) and increased 24 h after CPB to 270.9 pmol/min (*p* = 0.0024) (Table 2, Figures 3A,B). Maximum respiration (90.98 vs 60.03 pmol/min, *p* = 0.0002) and spare respiratory capacity (71.43 vs 48.64 pmol/min, *p* = 0.0001) had a transient dip 4 h after CPB (Table 2, Figures 3D,E). Proton leak and non-mitochondrial respiration showed no signifikant changes during surgery (Table 2, Figures 3F,G).

**Table 2:** Cell metabolism.

**Parameter**	**Controls [pmol/min]**	**Time point**	**Mean (95% CI) [pmol/min]**	***p* to t0**	***p* to controls**
ATP production	24.97 (13.12–30.22)	t0	12.95 (9.818–20.61)	-	0.0181
		t4	10.13 (8.138–13.71)	0.0024	<0.0001
		t24	17.26 (14.25–21.79)	n.s.	n. s.
Basal glycolysis	97.4 (59.13–144.6)	t0	179.2 (138.0–205.1)	-	0.0036
		t4	179.0 (156.8–211.1)	n.s.	0.0005
		t24	250.1 (194.5–300.2)	<0.0001	<0.0001
Compensatory glycolysis	124.8 (92.73–157.5)	t0	189.1 (152.9–211.2)	-	0.0031
		t4	186.6 (164.2–211.2)	n.s.	0.0018
		t24	270.9 (211.8–319.2)	0.0002	<0.00001
Basal respiration	25.07 (17.03–31.71)	t0	16.68 (14.28–20.91)	-	0.0294
		t4	13.25 (10.82–16.45)	0.0024	<0.0001
		t24	19.78 (17.08–24.29)	n.s.	n.s.
Maximum respiration	100.8 (87.62–116.9)	t0	90.98 (78.15–100.1)	-	n.s.
		t4	60.03 (54.4–77.2)	0.0002	<0.0001
		t24	98.27 (85.86–123.9)	n.s.	n.s.
Spare Respiratory Capacity	76.5 (64.66–99.82)	t0	71.43 (63.69–82.39)	-	n.s.
		t4	48.64 (36.55–61.29)	0.0001	>0.0001
		t24	79.64 (64.62–100.1)	n.s.	n.s.
Non-mitochondrial respiration	14.91 (13.38–16.21)	t0	15.45 (13.66–17.66)	-	n.s.
		t4	14.92 (12.53–17.4)	n.s.	n.s.
		t24	18.47 (16.27–20.75)	n.s.	0.0056
Proton leak	2.54 (0.3075–3.098)	t0	3.565 (2.225–4.183)	-	n.s.
		t4	2.835 (1.205–4.153)	n.s.	n.s.
		t24	2.83 (1.458–4.46)	n. s.	n.s.

*MWU test was used to test for significance; ATP, adenosine triphosphate; CI, confidence interval; t0, before surgery; t4, 4 h after the start of cardiopulmonary bypass (CPB); t24, 24 h after the start of CPB*.

Basal respiration in healthy individuals was significantly higher than in patients (25.07 vs. 16.68 pmol/min; *p* = 0.0294). The basal respiration rate decreased between baseline (16.68 pmol/min) and t4 (13.25 pmol/min) and was elevated again at t24 (19.78 pmol/min; *p* = 0.0024) (Table 2, Figures 3C,H).

### Plasma Cytokines and *ex vivo* Stimulation

**Figure 4:**
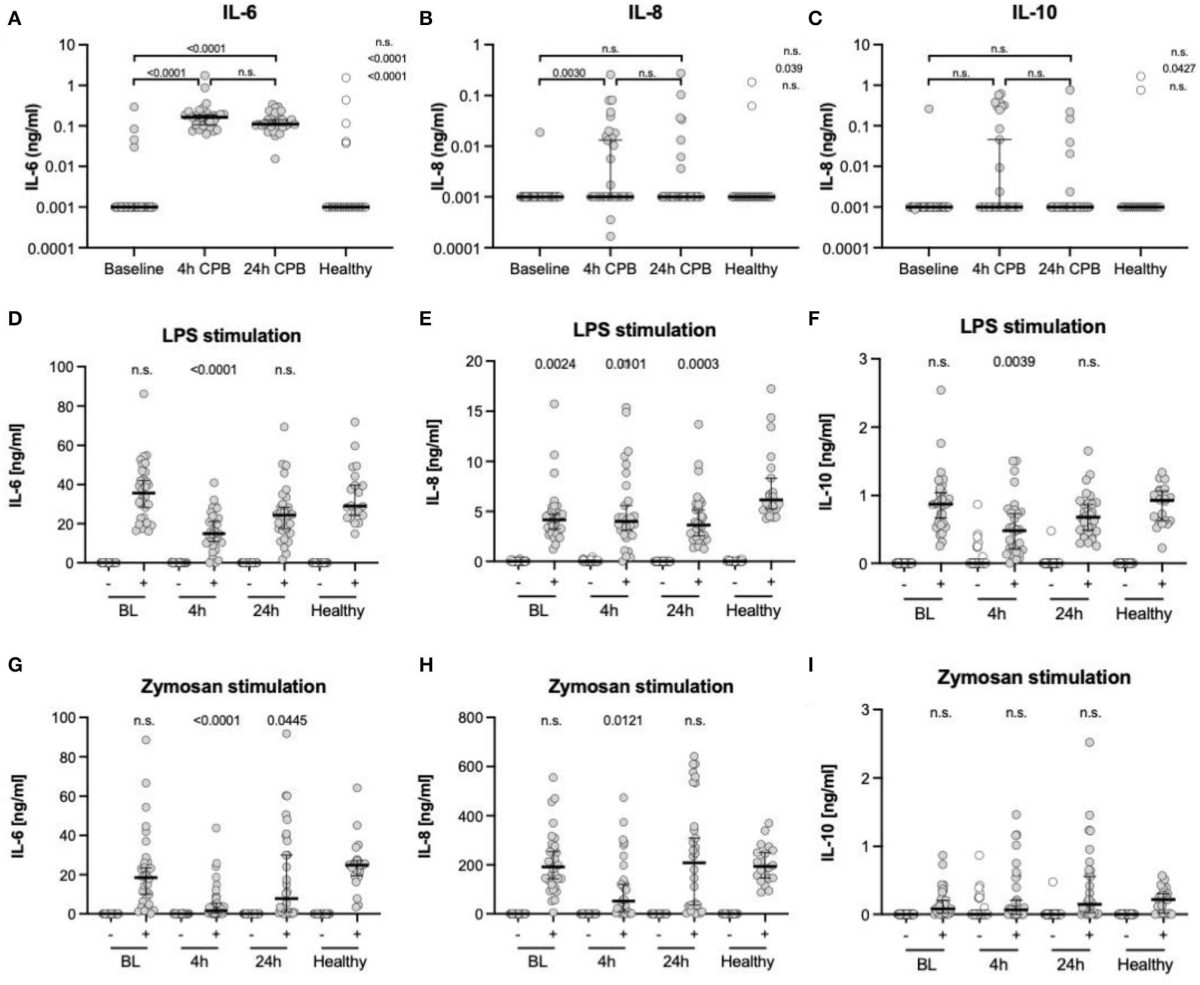
Cytokine levels in plasma and after *ex vivo* stimulation. ELISA analyses of cytokine levels in plasma **(A–C)**, after *ex vivo* stimulation with LPS **(D–F)** and zymosan **(G–I)**; all measurements were taken from blood samples before surgery (BL), 4 h after CPB (4 h), 24 h after CPB (24 h), and compared among each other and to the healthy control group; the *y*-axis in **(A–C)** is presented in a log scale and zero data has been set to 0.001; cytokine levels after stimulation were only compared to controls **(D–I)**; data shown as median and the whiskers of the 5th and 95th percentile; MWU test was used to test for significance; CPB, cardiopulmonary bypass; IL, interleukins.

We found an increase in the pro-inflammatory cytokines IL-6 and IL-8 4 h after CPB and a further decrease 24 h after CPB for IL-6 and IL-8 (Table 3, Figures 4A–C). The anti-inflammatory cytokine IL-10 showed no significant change.

**Table 3:** Cytokines and *ex vivo* whole-blood stimulation.

**Parameter**	**Controls [ng/ml]**	**Time point**	**Mean [ng/ml]**	**95% CI [ng/ml]**	***p* to t0**	***p* to controls**
**Plasma cytokines**						
IL6	0 (0–0)	t0	0 (0–0)		-	n. s.
		t4	0.16 (0.1–0.197)		<0.0001	<0.0001
		t24	0.11 (0.1–0.16)		<0.0001	<0.0001
IL8	0 (0–0)	t0	0 (0–0)		-	n. s.
		t4	0 (0–0.02)		0.003	0.039
		t24	0 (0–0)		n. s.	n. s.
IL10	0 (0–0)	t0	0 (0–0)		-	n. s.
		t4	0 (0–0.26)		n. s.	0.0427
		t24	0 (0–0)		n. s.	n. s.
**Cytokines after 24 h LPS stimulation**						
IL6	29.13 (16.25–31)	t0	35.68 (22.17–46.57)		-	n. s.
		t4	15.02 (10.25–24.78)		-	<0.0001
		t24	24.40 (16.25–31)		-	n. s.
IL8	6.18 (5.18–9.01)	t0	4.17 (2.98–5.12)		-	0.0024
		t4	4.13 (2.85–8.23)		-	0.0101
		t24	3.64 (2.2–5.73)		-	0.0003
IL10	0.93 (0.61–1.08)	t0	0.87 (0.59–1.11)		-	n. s.
		t4	0.49 (0.18–0.77)		-	0.0039
		t24	0.68 (0.45–0.89)		-	n. s.
**Cytokines after 24 h zymosan stimulation**						
IL6	24.86 (19.05–27.59)	t0	18.57 (6.01–27.04)		-	n. s.
		t4	1.65 (0.33–8.86)		-	<0.0001
		t24	7.91 (0.51–37.87)		-	0.0445
IL8	193.45 (145.61–257.47)	t0	191.49 (116.74–278.94)		-	n. s.
		t4	57.95 (7.62–168.66)		-	0.0121
		t24	208,523 (28,353–400,418)		-	n. s.
IL10	0.22 (0.02–0.36)	t0	0.08 (0.009–0.26)		-	n. s.
		t4	0.07 (0–0.55)		-	n. s.
		t24	0.15 (0.01–0.64)		-	n. s.

*MWU test was used to test for significance; CI, confidence interval; IL, interleukin; t0, before surgery; t4, 4 h after the start of cardiopulmonary bypass (CPB); t24, 24 h after the start of CPB*.

After *ex vivo* stimulation with LPS, there was an increase in the cytokines in all groups (Table 3, Figures 4D–I). At t4, IL-6 levels were lower in patients than in the control group. The IL-8 levels at every point in time were lower than in stimulated healthy controls. IL-10 showed only at the 4 h time point a significant decline. After *ex vivo* stimulation with zymosan, there were higher or similar levels of the cytokines IL-6, IL-8, and IL-10 in healthy controls compared to patients. The CPB group showed at t4 lower IL-6 levels than the control group. In the same manner, the IL-8 level at the 4 h time point was lower than the healthy at baseline. The remaining IL-8 level was at no time significantly different from the control group at baseline. For IL-10, there was no significant and relevant change at any point in time compared to healthy controls.

### Fluorescence-Activated Cell Sorting

#### Human Leukocyte Antigen-DR Isotype

There was a significant decrease in HLA-DR expression 4 h after the start of the CPB, and this depression lasted at least till 24 h after the start of the CPB (Table 4, Figure 2C).

**Table 4:** Results of FACS: HLA-DR, PD-L1/2, and VISTA.

**Parameter**	**Controls [ΔMFI]**	**Time point**	**Mean [ΔMFI]**	**95% CI [ΔMFI]**	***p* to t0**	***p* to Controls**
HLA-DR	39,947 (34,505–44,250)	t0	35,177 (28,710–42,899)		-	n.s.
		t4	19,764 (15,909–24,933)		<0.0001	<0.0001
		t24	18,120 (14,696–22,386)		0.0001	<0.0001
PD-L1	1,005 (909.8–1,315)	t0	689.5 (560.3–1,022)		-	0.0092
		t4	727.5 (558.3–936.5)		n.s.	0.0042
		t24	679.5 (575.8–1,014)		n.s.	0.0076
PD-L2	133.5 (117.8–170)	t0	107 (90–145.5)		-	n.s.
		t4	56 (45.75–74.25)		<0.0001	<0.0001
		t24	108 (80–140)		n.s.	n.s.
VISTA	391.5 (326.5–472)	t0	257.5 (190–408)		-	0.0467
		t4	393.5 (243.3–526.3)		n.s.	n.s.
		t24	369.5 (297.8–421.5)		n.s.	n.s.

*MWU test was used to test for significance; FACS, fluorescence-activated cell sorting; CI, confidence interval; HLA, human leukocyte antigen system; ΔMFI, delta mean fluorescence intensity; PD-L, programmed cell death ligand; t0, before surgery; t4, 4 h after the start of cardiopulmonary bypass (CPB); t24, 24 h after the start of CPB; VISTA, V-domain Ig suppressor of T cell activation*.

#### Programmed Cell Death 1 Ligand, Programmed Cell Death 2 Ligand, and V-Domain Ig Suppressor of T Cell Activation

We found a significant decrease in PD-L2 expression at the 4 h time point (107 vs. 56 ΔMFI; *p* < 0.0001), but no changes in PD-L1 and VISTA expression in the course of surgery were seen. However, the preoperative VISTA and PD-L1 expression was reduced compared to the healthy individuals (258 vs. 392 ΔMFI; *p* = 0.0467) (257.5 vs. 391.5 ΔMFI; *p* = 0.0467, and 1,005 vs 689.5 ΔMFI; *p* = 0.0092) (Table 4, Figures 2D–F).

## Discussion

This observational study shows for the first time a change in immunometabolism during cardiac surgery. In the course of surgery, the monocytes display an increase in glycolysis and a transient decrease in OXPHOS, which is characteristic of aerobic glycolysis. The change of intracellular metabolic pathways in monocytes according to a Warburg effect can be led back to inflammatory stimuli during cardiac surgery and CPB.

An increase in the plasma cytokine levels of the inflammatory cytokines IL-6 and IL-8, as shown in this study, is typical for patients undergoing cardiac surgery (2, 3, 21). IL-6 can increase the expression of glycolytic enzymes and promote aerobic glycolysis in macrophages (22, 23). Likewise, IL-8 has been shown to mediate the enhancement of glycolysis (24). Pro- and anti-inflammatory cytokines are capable of changing the metabolism in immune cells in a variety of ways. The hypoxia-inducible factor 1α (HIF-1α) is a transcription factor and a strong inducer of glycolysis (25). Recent studies showed the effects of commonly used anesthetics, such as sevoflurane, on increasing HIF-1α and thereby aerobic glycolysis (26). Even though anesthesia has an impact on inflammation and immunometabolism, it is one part of a complex arrangement of immunological stimuli in cardiac surgery using CPB. All patients included in our study have received a comparable regimen of anesthetics and halogenated gases for the maintenance of anesthesia. In this study, we therefore consider anesthesia as an indelible part of cardiac surgery using CPB. Besides hypoxia, HIF-1α can be activated by the cytokines IL-6, TNF-α, and IL-1β, which are elevated during cardiac surgery using CPB (1–3, 27, 28). Dendritic cells and macrophages are primarily using aerobic glycolysis after being stimulated by proinflammatory cytokines, such as interferons (29, 30). The anti-inflammatory cytokine IL-10 can regulate aerobic glycolysis in macrophages by preserving OXPHOS (31). IL-10 showed no changes in this study, which could be explained by the early points in time measuring. In previous studies, a peak of IL-10 was detected at the third postoperative day (32). A significant increase in IL-10 after injury only occurs in a few cases and needs a severe trauma (33). In addition, some authors postulate a genetic predisposition to be a relevant influencing factor on the cytokine response after cardiac surgery (34).

Immunometabolism is also modulated by the complement system and Toll-like receptors (TLRs) *via* DAMPS, which are inevitably released during surgery (4, 7). The stimulation of TLRs results in enhanced glycolysis and depending on TLR type in an increase or decrease of OXPHOS (25). Besides DAMPS, exogenous molecules are known to activate monocytes and initiate metabolic changes. LPS of bacteria is elevated in the bloodstream during cardiac surgery, which is probably caused by transmission of gut bacteria through dysfunctional barriers of the intestine during CPB (35, 36). Especially LPS has been reliably used to generate aerobic glycolysis in monocytes (25). Hypoxia is a strong inducer of glycolysis *via* HIF-1α. To prevent ischemia, perioperative oxygen levels are usually kept above physiological values during cardiac surgery (11). Although an extensive and frequent systemic hypoxia during cardiac surgery seems to be unlikely, local ischemia (e.g., of the myocardium) due to aortic clamping is not (37) and could lead to metabolic changes in monocytes.

The change in monocytes in the course of surgery can also be seen in their expression of surface markers, such as the observed decrease in HLA-DR and PD-L2. This effect has been detected during and after cardiac surgery procedures using CPB, as well as after severe trauma or during sepsis (19, 38, 39). The HLA-DR downregulation is linked to an inhibition of OXPHOS and is associated with complications (such as infections) as a sign of monocyte anergy (40, 41). This is consistent with a lower IL-6 and IL-8 response to LPS or zymosan stimulation at this point in time. Decreased monocytic function and cytokine levels after full blood stimulation have been observed in pediatric cardiac surgery with CPB (42). However, the percentage of CD14^+^ monocytes decreases 4 h after CPB, which could mask a trained immunity with high cytokine levels after *ex vivo* stimulation. The reduction in PD-L2 during surgery indicates higher inflammatory responses by immune cells and could explain elevated cytokine levels of IL-6 and IL-8 measured after CPB and at the same time a lower IL-10 release after LPS stimulation (43). Inflammatory effects and cytokine release are also associated with the suppression of PD-L1 and VISTA. Furthermore, the expression of PD-L1 is linked directly to the activity of HIF-1α and consequently to immunometabolism (20). Both were already downregulated in patients before surgery, with PD-L1 remaining low during surgery.

Surprisingly, this study detected a Warburg effect in monocytes of patients even before surgery, which can be seen compared to monocytes of healthy controls. This means that there has been a preexisting shift from OXPHOS to glycolysis in terms of a trained immunity with no acute trigger, such as surgery or hypoxia (44). Considering potential previous stimuli directly leads to common diseases in cardiac surgery patients, such as atherosclerosis. Monocytes of patients with atherosclerosis showed a higher glycolytic rate and an increased mRNA expression of IL-6 and IL-1β compared to healthy controls (45). Although this has only been described after *in vitro* stimulation (with LPS and INF-γ), monocytes of atherosclerotic patients seem to be pre-exposed to activating stimuli. Riksen et al. propose that long-time changes in monocytes are caused by changes in bone marrow progenitors and are leading to epigenetic and metabolic modifications in the form of trained immunity, which are important factors in atherosclerosis (46). Increased levels of pro-atherosclerotic markers, such as lipoprotein (a) and oxidized low-density lipoprotein (oxLDL), already affect glycolysis in progenitor cells and can set off aggravated long-term immune responses in monocytes (47–49). In patients with symptomatic coronary artery disease, monocytes showed an increased immune response to LPS and RNA analysis of whole blood, suggesting a high glycolytic rate, compatible with aerobic glycolysis (50).

## Limitations

This study is an observation trial with an explorative design. We cannot demonstrate any causality. Because of the many influencing factors, it is difficult and not implemented to match the patients and the healthy volunteers according to all potentially influencing factors of metabolism, such as comorbidities. For instance, atherosclerosis, including coronary heart disease, is an inflammatory disease of the arterial wall. We excluded patients with diagnosed diabetes mellitus, but there is the possibility of underdetection and prediabetes. Moreover, we hereby excluded a large proportion of patients undergoing cardiac surgery because of the common occurrence of this illness in the cardiovascular patient collective. Also, we excluded controls receiving statin treatment because of the potential influence on cell metabolism (45). But hyperlipidemia is a frequent comorbidity of patients undergoing cardiac surgery and therefore is a statin treatment. In this context, different authors have shown that some commonly used drugs, such as aspirin, metoprolol, lisinopril, and simvastatin, can lower a trained immunity in macrophages (34) and statins also prevent a trained immunity of monocytes *in vivo* (39).

The stimulation with LPS and zymosan was performed on full blood, with individual variations in the number of monocytes. This could impair the comparability between patients and make it difficult to evaluate the extent of monocytic cytokine response.

Because of methodical reasons, we did not differentiate between different monocyte subpopulations; consequently, we could not make any statement about the influence of the different populations. This influence on the inflammatory reaction is described by different authors (51, 52). In respect thereof, it is likely that this change in monocyte population had an influence on the measurements we made during our trial.

## Conclusion

Our data suggest that in the typical patient undergoing cardiac surgery collective is a switch from OXPHOS to aerobic glycolysis compared to healthy individuals. After surgery and usage of a CPB, there have already been described multicausal inflammatory reactions. Furthermore, there is a shift in metabolism favoring aerobic glycolysis during the examined 24 h after CPB. Both effects seem equivalent to the Warburg effect, which has been described in different cell types. These findings extend the understanding of inflammation in patients undergoing cardiac surgery and could reveal new therapeutic approaches before and during surgery. Further research will be necessary to ensure the mechanisms for the switch in metabolism and its pathophysiological implications.

## Data Availability Statement

The raw data supporting the conclusions of this article will be made available by the authors, without undue reservation.

## Ethics Statement

The studies involving human participants were reviewed and approved by Ethics Committee of the Medical Faculty of the Heidelberg University (Az: S-112/2018). The patients/participants provided their written informed consent to participate in this study.

## Author Contributions

MA, DM, FU, and CL were responsible for study design and conduct, data and sample acquisition, statistical analysis, and interpretation as well as for writing the manuscript. RA and JS acquired samples and data, assisted in the statistical analysis and interpretation, and approved and helped to draft the manuscript. FL, MK, and MW supported the study conduct as well as data acquisition, discussed the data and participated in data interpretation as well as manuscript preparation. All authors read and approved the final version of the manuscript.

## Funding

This study was supported by an grant of Heidelberger Stiftung Chirurgie, Heidelberg, Germany.

## Conflict of Interest

The authors declare that the research was conducted in the absence of any commercial or financial relationships that could be construed as a potential conflict of interest.

## Publisher's Note

All claims expressed in this article are solely those of the authors and do not necessarily represent those of their affiliated organizations, or those of the publisher, the editors and the reviewers. Any product that may be evaluated in this article, or claim that may be made by its manufacturer, is not guaranteed or endorsed by the publisher.

## Abbreviations

ATP: Adenosine triphosphate
BSA: Bovine serum albumin
CPB: Cardiopulmonary bypass
DAMPs: Damage-associated molecular patterns
DPBS: Dulbecco's phosphate-buffered saline solution
ECAR: Extracellular acidification rate
EDTA: Ethylenediaminetetraacetic acid
ELISA: Enzyme-linked immunosorbent assay
FACS: Fluorescence-activated cell sorting
FCCP: Carbonyl cyanide-p-trifluoromethoxyphenylhydrazone
FITC: Fluorescein isothiocyanate
FMO: Fluorescence minus one
FSC: Forward Scatter
HEPES: 4-(2-hydroxyethyl)-1-piperazineethanesulfonic acid
HLA-DR: Human leukocyte antigen–DR isotype
HIF-1α: Hypoxia-inducible factor 1α
IL: Interleukin
LPS: Lipopolysaccharide
MACS: Magnetic-activated cell sorting
MWU: Mann-Whitney *U*
MFI: Mean fluorescence intensity
MODS: Multiorgan dysfunction syndrome
OXPHOS: Oxidative phosphorylation
oxLDL: Oxidized low-density lipoprotein
OCR: Oxygen consumption rate
PD-L: Programmed cell death 1 ligand
PER: Proton efflux rate
PMBCs: Peripheral blood mononuclear cells
RT: Room temperature
SIRS: Systemic inflammatory response syndrome
SSC: Side scatter
t0: Before the start of CPB
t4: Four hours after the start of CPB
t24: Twenty-four hours after the start of CPB
TNF- α: Tumor necrosis factor alpha
TLRs: Toll-like receptors
VISTA: V-domain Ig suppressor of T cell activation.

## References

[1] Meldrum DR Partrick DA Cleveland JC Jr Shenkar R Meldrum KK Raiesdana A. On-pump coronary artery bypass surgery activates human myocardial NF-kappaB and increases TNF-alpha in the heart. J. Surg. Res. (2003) 112:175–9. 10.1016/S0022-4804(03)00122-7 12888335

[2] Wan S Yim AP. Cytokines in myocardial injury: impact on cardiac surgical approach. Eur J Cardiothorac Surg. (1999) 16 Suppl 1:S107–11. 10.1016/S1010-7940(99)00200-6 10536961

[3] Wan S LeClerc JL Vincent JL. Cytokine responses to cardiopulmonary bypass: lessons learned from cardiac transplantation. Ann Thorac Surg. (1997) 63:269–76. 10.1016/S0003-4975(96)00931-9 8993291

[4] Chenoweth DE Cooper SW Hugli TE Stewart RW Blackstone EH Kirklin JW. Complement activation during cardiopulmonary bypass: evidence for generation of C3a and C5a anaphylatoxins. N Engl J Med. (1981) 304:497–503. 10.1056/NEJM198102263040901 7453783

[5] Dabrowska AM Slotwinski R. The immune response to surgery and infection. Cent Eur J Immunol. (2014) 39:532–7. 10.5114/ceji.2014.47741 26155175 PMC4439968

[6] Czerny M Baumer H Kilo J Lassnigg A Hamwi A Vukovich T. Inflammatory response and myocardial injury following coronary artery bypass grafting with or without cardiopulmonary bypass. Eur J Cardiothorac Surg. (2000) 17:737–42. 10.1016/S1010-7940(00)00420-6 10856869

[7] Cavarocchi NC Schaff HV Orszulak TA Homburger HA Schnell WA Jr Pluth JR. Evidence for complement activation by protamine-heparin interaction after cardiopulmonary bypass. Surgery. (1985) 98:525–31. 3875906

[8] Hoel TN Videm V Mollnes TE Saatvedt K Brosstad F Fiane AE. Off-pump cardiac surgery abolishes complement activation. Perfusion. (2007) 22:251–6. 10.1177/0267659107084142 18181513

[9] Kirklin JK Westaby S Blackstone EH Kirklin JW Chenoweth DE Pacifico AD. Complement and the damaging effects of cardiopulmonary bypass. J Thorac Cardiovasc Surg. (1983) 86:845–57. 10.1016/S0022-5223(19)39061-0 6606084

[10] Laffey JG Boylan JF Cheng DC. The systemic inflammatory response to cardiac surgery: implications for the anesthesiologist. Anesthesiology. (2002) 97:215–52. 10.1097/00000542-200207000-00030 12131125

[11] Spoelstra-de Man AM Smit B Oudemans-van Straaten HM Smulders YM. Cardiovascular effects of hyperoxia during and after cardiac surgery. Anaesthesia. (2015) 70:1307–19. 10.1111/anae.13218 26348878

[12] Clermont G Vergely C Jazayeri S Lahet JJ Goudeau JJ Lecour S. Systemic free radical activation is a major event involved in myocardial oxidative stress related to cardiopulmonary bypass. Anesthesiology. (2002) 96:80–7. 10.1097/00000542-200201000-00019 11753006

[13] Palsson-McDermott EM O'Neill LA. The Warburg effect then and now: from cancer to inflammatory diseases. Bioessays. (2013) 35:965–73. 10.1002/bies.201300084 24115022

[14] McGettrick AF O'Neill LA. How metabolism generates signals during innate immunity and inflammation. J Biol Chem. (2013) 288:22893–8. 10.1074/jbc.R113.486464 23798679 PMC3743468

[15] Warburg O Wind F Negelein E. The metabolism of tumors in the body. J Gen Physiol. (1927) 8:519–30. 10.1085/jgp.8.6.519 19872213 PMC2140820

[16] van Furth R. Human monocytes and cytokines. J Immunol Res. (1998) 149:719–20. 10.1016/S0923-2494(99)80045-5 9851530

[17] Ziegler-Heitbrock L. Blood monocytes and their subsets: established features and open questions. Front. Immunol. (2015) 6:423. 10.3389/fimmu.2015.00423 26347746 PMC4538304

[18] O'Neill LA Kishton RJ Rathmell J. A guide to immunometabolism for immunologists. Nat Rev Immunol. (2016) 16:553–65. 10.1038/nri.2016.70 27396447 PMC5001910

[19] Franke A Lante W Zoeller LG Kurig E Weinhold C Markewitz A. Delayed recovery of human leukocyte antigen-DR expression after cardiac surgery with early non-lethal postoperative complications: only an epiphenomenon? Interact Cardiovasc Thorac Surg. (2008) 7:207–11. 10.1510/icvts.2007.158899 18055481

[20] Barsoum IB Smallwood CA Siemens DR Graham CH. A mechanism of hypoxia-mediated escape from adaptive immunity in cancer cells. Cancer research. (2014) 74:665–74. 10.1158/0008-5472.CAN-13-0992 24336068

[21] Wei M Kuukasjarvi P Laurikka J Pehkonen E Kaukinen S Laine S. Cytokine responses in low-risk coronary artery bypass surgery. Int J Angiol. (2001) 10:27–30. 10.1007/BF01616340 11178783

[22] Zhang Y Yu G Chu H Wang X Xiong L Cai G. Macrophage-associated PGK1 phosphorylation promotes aerobic glycolysis and tumorigenesis. Molecular Cell. (2018) 71:201–15.e7. 10.1016/j.molcel.2018.06.023 30029001

[23] Ando M Uehara I Kogure K Asano Y Nakajima W Abe Y. Interleukin 6 Enhances Glycolysis through Expression of the Glycolytic Enzymes Hexokinase 2 and 6-Phosphofructo-2-kinase/Fructose-2,6-bisphosphatase-3. J Nippon Med Sch. (2010) 77:97–105. 10.1272/jnms.77.97 20453422

[24] Xu H Zeng Y Liu L Gao Q Jin S Lan Q. PRL-3 improves colorectal cancer cell proliferation and invasion through IL-8 mediated glycolysis metabolism. Int J Oncol. (2017) 51:1271–9. 10.3892/ijo.2017.4090 28791350

[25] Lachmandas E Boutens L Ratter JM Hijmans A Hooiveld GJ Joosten LA. Microbial stimulation of different Toll-like receptor signalling pathways induces diverse metabolic programmes in human monocytes. Nat Microbiol. (2016) 2:16246. 10.1038/nmicrobiol.2016.246 27991883

[26] Zhou T Guo S Wang S Li Q Zhang M. Protective effect of sevoflurane on myocardial ischemia-reperfusion injury in rat hearts and its impact on HIF-1α and caspase-3 expression. Exp Ther Med. (2017) 14:4307–11. 10.3892/etm.2017.5078 29104643 PMC5658739

[27] Pearce EL Pearce EJ. Metabolic pathways in immune cell activation and quiescence. Immunity. (2013) 38:633–43. 10.1016/j.immuni.2013.04.005 23601682 PMC3654249

[28] Fu X Zhai S Yuan J. Interleukin-6 (IL-6) triggers the malignancy of hemangioma cells via activation of HIF-1α/VEGFA signals. Eur J Pharmacol. (2018) 841:82–9. 10.1016/j.ejphar.2018.10.022 30342949

[29] Martinez FO Sica A Mantovani A Locati M. Macrophage activation and polarization. Front Biosci. (2008) 13:453–61. 10.2741/2692 17981560

[30] Pantel A Teixeira A Haddad E Wood EG Steinman RM Longhi MP. Direct type I IFN but not MDA5/TLR3 activation of dendritic cells is required for maturation and metabolic shift to glycolysis after poly IC stimulation. PLoS Biol. (2014) 12:e1001759. 10.1371/journal.pbio.1001759 24409099 PMC3883643

[31] Baseler WA Davies LC Quigley L Ridnour LA Weiss JM Hussain SP. Autocrine IL-10 functions as a rheostat for M1 macrophage glycolytic commitment by tuning nitric oxide production. Redox Biol. (2016) 10:12–23. 10.1016/j.redox.2016.09.005 27676159 PMC5037266

[32] Roth-Isigkeit A Borstel TV Seyfarth M Schmucker P. Perioperative serum levels of tumour-necrosis-factor alpha (TNF-alpha), IL-1 beta, IL-6, IL-10 and soluble IL-2 receptor in patients undergoing cardiac surgery with cardiopulmonary bypass without and with correction for haemodilution. Clin Exp Immunol. (1999) 118:242–6. 10.1046/j.1365-2249.1999.01050.x 10540185 PMC1905422

[33] Golabek-Dropiewska K Pawlowska J Witkowski J Lasek J Marks W Stasiak M. Analysis of selected pro- and anti-inflammatory cytokines in patients with multiple injuries in the early period after trauma. Cent Eur J Immunol. (2018) 43:42–9. 10.5114/ceji.2018.74872 29731691 PMC5927172

[34] Roth-Isigkeit A Hasselbach L Ocklitz E Bruckner S Ros A Gehring H. Inter-individual differences in cytokine release in patients undergoing cardiac surgery with cardiopulmonary bypass. Clin Exp Immunol. (2001) 125:80–8. 10.1046/j.1365-2249.2001.01521.x 11472429 PMC1906109

[35] Andersen LW Landow L Baek L Jansen E Baker S. Association between gastric intramucosal pH and splanchnic endotoxin, antibody to endotoxin, and tumor necrosis factor-alpha concentrations in patients undergoing cardiopulmonary bypass. Crit Care Med. (1993) 21:210–7. 10.1097/00003246-199302000-00011 8428471

[36] Tao W Zwischenberger JB Nguyen TT Vertrees RA McDaniel LB Nutt LK. Gut mucosal ischemia during normothermic cardiopulmonary bypass results from blood flow redistribution and increased oxygen demand. J Thorac Cardiovasc Surg. (1995) 110:819–28. 10.1016/S0022-5223(95)70116-8 7564451

[37] Corcoran SE O'Neill LA. HIF1alpha and metabolic reprogramming in inflammation. J Clin Invest. (2016) 126:3699–707. 10.1172/JCI84431 27571407 PMC5096812

[38] Kim OY Monsel A Bertrand M Coriat P Cavaillon JM Adib-Conquy M. Differential down-regulation of HLA-DR on monocyte subpopulations during systemic inflammation. Critical Care. (2010) 14:R61. 10.1186/cc8959 20385017 PMC2887183

[39] McBride WT Armstrong MA Crockard AD McMurray TJ Rea JM. Cytokine balance and immunosuppressive changes at cardiac surgery: contrasting response between patients and isolated CPB circuits. Br J Anaesth. (1995) 75:724–33. 10.1093/bja/75.6.724 8672321

[40] Venet F Lepape A Monneret G. Clinical review: flow cytometry perspectives in the ICU - from diagnosis of infection to monitoring of injury-induced immune dysfunctions. Critical Care. (2011) 15:231. 10.1186/cc10333 22017882 PMC3334725

[41] Krauß PL Buttgereit F Gaber T Pfeiffenberger M Chen Y Buttgereit T. AB0029 the metabolic hierarchy of immune processes in human monocytes. Ann Rheum Dis. (2021) 80:1048. 10.1136/annrheumdis-2021-eular.79433903093

[42] Justus G Walker C Rosenthal LM Berger F Miera O Schmitt KRL. Immunodepression after CPB: cytokine dynamics and clinics after pediatric cardiac surgery-a prospective trial. Cytokine. (2019) 122:154018. 10.1016/j.cyto.2017.03.017 28411047

[43] Sharpe AH Freeman GJ. The B7-CD28 superfamily. Nat Rev Immunol. (2002) 2:116–26. 10.1038/nri727 11910893

[44] van der Meer JW Joosten LA Riksen N Netea MG. Trained immunity: a smart way to enhance innate immune defence. Mol Immunol. (2015) 68:40–4. 10.1016/j.molimm.2015.06.019 26597205

[45] Shirai T Nazarewicz RR Wallis BB Yanes RE Watanabe R Hilhorst M. The glycolytic enzyme PKM2 bridges metabolic and inflammatory dysfunction in coronary artery disease. J Exp Med. (2016) 213:337–54. 10.1084/jem.20150900 26926996 PMC4813677

[46] Riksen NP. Trained immunity and atherosclerotic cardiovascular disease. Curr Opin Lipidol. (2019) 30:395–400. 10.1097/MOL.0000000000000628 31335332

[47] Groh L Keating ST Joosten LAB Netea MG Riksen NP. Monocyte and macrophage immunometabolism in atherosclerosis. Semin Immunopathol. (2018) 40:203–14. 10.1007/s00281-017-0656-7 28971272 PMC5809534

[48] Bekkering S Quintin J Joosten LA van der Meer JW Netea MG Riksen NP. Oxidized low-density lipoprotein induces long-term proinflammatory cytokine production and foam cell formation via epigenetic reprogramming of monocytes. Arterioscler Thromb Vasc Biol. (2014) 34:1731–8. 10.1161/ATVBAHA.114.303887 24903093

[49] Sarrazy V Viaud M Westerterp M Ivanov S Giorgetti-Peraldi S Guinamard R. Disruption of glut1 in hematopoietic stem cells prevents myelopoiesis and enhanced glucose flux in atheromatous plaques of ApoE(-/-) Mice. Circ Res. (2016) 118:1062–77. 10.1161/CIRCRESAHA.115.307599 26926469 PMC4824305

[50] Bekkering S van den Munckhof I Nielen T Lamfers E Dinarello C Rutten J. Innate immune cell activation and epigenetic remodeling in symptomatic and asymptomatic atherosclerosis in humans *in vivo*. Atherosclerosis. (2016) 254:228–36. 10.1016/j.atherosclerosis.2016.10.019 27764724

[51] Patel AA Zhang Y Fullerton JN Boelen L Rongvaux A Maini AA. The fate and lifespan of human monocyte subsets in steady state and systemic inflammation. J Exp Med. (2017) 214:1913–23. 10.1084/jem.20170355 28606987 PMC5502436

[52] Valencia-Nunez DM Kreutler W Merino A Muñoz-Carvajal I Holzhey D Aljama P. Acute monocyte subset counts in patients undergoing coronary surgery. Clin Surg. (2018) 3:2010.

